# Multi-trait selection defines a plant-architecture ideotype for soybean in the Russian Far East

**DOI:** 10.3389/fpls.2026.1922888

**Published:** 2026-09-04

**Authors:** Yawar Habib, Tatiana Aseeva, Oksana Shepel, Elena Potokina

**Affiliations:** 1 Center for Bio and Medical Technologies, Skolkovo Institute of Science and Technology, Moscow, Russia; 2 Far Eastern Research Institute of Agriculture, Khabarovsk Federal Research Center, Far East Branch, Russian Academy of Sciences, Vostochnoe, Russia; 3 Institute of Forest and Natural Resources Management, Saint Petersburg State Forest Technical University, Saint Petersburg, Russia

**Keywords:** early maturity, ideotype, multi-trait selection, plant architecture, Russian Far East, soybean

## Abstract

Soybean production in the Russian Far East is constrained by a short frost-free growing period, making early-maturing, productive cultivars a major regional breeding objective. Plant architecture influences the number of pod-bearing sites and the efficiency of mechanical harvesting, yet no architectural ideotype has been defined for this region and the genotypes closest to such a target are unknown. Architectural traits are correlated with one another and with maturity, and must therefore be selected simultaneously. In this study, 63 soybean genotypes were evaluated over three years (2018–2020) at a single site in Khabarovsk Krai, Russia (48°N). Plant height, first pod height, number of branches, productive nodes on the main stem and on lateral branches, and days to maturity were recorded. All six traits showed significant genotypic variation, moderate to high broad-sense heritability (0.65–0.91), and a significant genotype by year interaction. The two branching traits combined high heritability with the highest genetic advance (67.3% and 78.2% of the mean). Correlation and principal component analyses revealed two independent dimensions, one combining plant size with later maturity and one representing branching. An ideotype was defined according to regional priorities, combining earlier maturity with a taller, more productive plant structure suited to mechanical harvesting. The multi-trait genotype-ideotype distance index (MGIDI) selected the nine genotypes closest to this target, with selection differentials in the desired direction for five of the six traits. The number of productive nodes on the main stem was the only trait to move against its desired direction, because it is genetically associated with later maturity. The multi-trait stability index (MTSI) selected nine genotypes on mean performance and year-to-year consistency, with selection differentials in the desired direction for all six traits. Gaterslebener stamm, VNIIS 1, ISZ 16, and No. 700 were selected by both indices, with Gaterslebener stamm ranking first under both. The stability estimated here is temporal, reflecting year-to-year consistency rather than adaptation across locations. These four genotypes are recommended for multi-location evaluation and as parents for breeding early-maturing cultivars suited to mechanical harvesting.

## 1 Introduction

Soybean (*Glycine max* (L.) Merr.) provides nearly 60% of global oilseed production (Duan et al., 2023) and over two-thirds of world protein-meal output (USDA-FAS, 2025). Global production rose by approximately 50% between 2010 and 2024, largely through the expansion of cultivated area (FAO, 2025). Sustaining this role requires continued genetic improvement in yield and adaptation, particularly in the climatically marginal regions into which cultivation is expanding. The Russian Far East is one such region and a long-established centre of soybean cultivation in Russia. National soybean area has more than doubled over the past decade to approximately 4 million hectares, and the 2023 harvest reached a record 6.6 million tonnes (USDA-FAS, 2025). The Far Eastern Federal District accounts for about 38% of national production, second only to the Central Federal District (Volkova and Smolyaninova, 2023). Soybean breeding for the region is conducted by a small number of institutions, including the Far Eastern Research Institute of Agriculture (FERIA) in Khabarovsk Krai, which has released locally adapted cultivars (Sinegovskaya, 2021). The region has a monsoonal, humid-continental climate, whose short frost-free period and high interannual variability in temperature and growing-season precipitation impose severe constraints on the crop.

Early maturity is therefore the principal breeding priority for soybean at these high latitudes. Time to flowering and maturity is a strongly photoperiodic trait, and adaptation to long-day, high-latitude environments is governed largely by allelic variation at the maturity loci *E1–E4*, of which *E1* is the central repressor of flowering under long days (Lin et al., 2021). Recessive, loss-of-function alleles across these loci progressively weaken photoperiod sensitivity, and their combinations produce the early-maturing, photoperiod-insensitive germplasm capable of flowering and ripening before the onset of early frost (Jia et al., 2014; Jiang et al., 2014). Selection at the homoeologous *PSEUDO-RESPONSE REGULATOR* genes *Tof11* and *Tof12* during domestication further shortened the growth period and extended cultivation to higher latitudes (Lu et al., 2020). Breeding for the high-latitude cold regions of Northeast Asia, including the Russian Far East, has consequently emphasised very early maturity groups (Jia et al., 2014).

Beyond phenology, the architecture of the soybean plant is a key determinant of yield potential and adaptation, and is increasingly recognised as an important target for further improvement (Li et al., 2024). Plant height, branch number, and branch angle shape canopy closure and light interception (Sreekanta et al., 2024; Virdi et al., 2023). Branch and node number determine the number of pod-bearing sites that ultimately set yield (Xu et al., 2021). Architectural traits also affect the practicality of cultivation. The height of the lowest pod determines how efficiently a crop can be recovered by combine harvesting, since pods borne below the cutter bar are lost (Kuzbakova et al., 2022). Defining the architecture best suited to a given production system is thus a breeding goal in its own right. The optimum for each trait, however, is environment-specific. Plant height is conventionally kept low to limit lodging in high-yielding, high-input systems (Hwang and Lee, 2019). This constraint is relaxed under the short-season, moderate-density conditions of the Russian Far East, where a taller, more pod-bearing stature may instead be advantageous. A further constraint is that larger plants generally mature later. Architectural size is therefore difficult to reconcile with the early maturity demanded at high latitude, where the breeding challenge is to shorten the growth cycle whilst preserving sufficient vegetative growth to support yield (Jia et al., 2014; Copley et al., 2018). Combining such antagonistic objectives within a single plant type requires an explicitly multi-trait approach.

The ideotype concept provides the basis for such an approach. An ideotype is an idealised plant type expected to perform well in a target environment (Donald, 1968). Selection is directed towards it, rather than allowing superior types to emerge only from yield testing. The framework remains widely applied in crop improvement, both to design future cultivars and to organise selection across many traits (Rötter et al., 2015; Carbajal-Friedrich and Burgess, 2024). Realising an ideotype requires selecting on several correlated traits simultaneously. Single-trait selection is poorly suited to this, because improving one trait in isolation may compromise others. This problem motivated the development of multi-trait selection indices (Hazel, 1943). Later formulations set selection targets directly rather than through economic weights, as in the desired-gain and genomic selection indices applied in other crops (Habib et al., 2026). In the present study the ideotype is defined operationally. Each trait is assigned a desired direction of change rather than an absolute target value, so that genotypes can be ranked by their multivariate distance from that target.

Two such indices have been developed within a single factor-analytic framework. The first is the multi-trait genotype-ideotype distance index (MGIDI), which ranks genotypes by their distance from a user-defined ideotype across all traits (Olivoto and Nardino, 2021). The factor analysis reduces the correlated traits to a smaller set of uncorrelated factors, and the economic weighting required by classical selection indices is avoided. The second is the multi-trait stability index (MTSI), which combines mean performance with stability across environments (Olivoto et al., 2019b). It incorporates the BLUP-based stability measure WAASB (Olivoto et al., 2019a) to identify genotypes that are both superior and consistent. In soybean, MGIDI has been applied to select for yield, early maturity, and adaptability (Maranna et al., 2021). The two indices have also been combined to integrate performance and stability (Ridara et al., 2026). Because MTSI estimates stability from the variation amongst test environments, its interpretation depends on whether those environments are spatial or temporal.

To our knowledge, these indices have not yet been applied to soybean in the Russian Far East. Regional germplasm has been characterised for agronomic performance and early maturity (Shepel et al., 2023), and candidate genes for flowering and maturity time have been identified by genome-wide association mapping (Perfil’ev et al., 2024). The architectural traits that determine yield potential and harvestability, however, have not been integrated into an explicit ideotype, and the genotypes closest to such a target remain unidentified. The present study addresses this gap by characterising the plant architecture of 63 soybean genotypes evaluated over three years (2018–2020) in the Russian Far East. Specifically, the objectives were to (i) quantify the phenotypic variation, genetic parameters, and trait interrelationships of five architectural traits and days to maturity; (ii) define an ideotype reflecting regional breeding priorities, combining earlier maturity with taller plants, a higher first pod, and more branches and productive nodes; and (iii) identify the genotypes closest to this ideotype, and the most temporally stable amongst them, using the MGIDI and MTSI selection indices.

## 2 Materials and methods

### 2.1 Experimental site, plant material, and trait measurement

**Figure 1:**
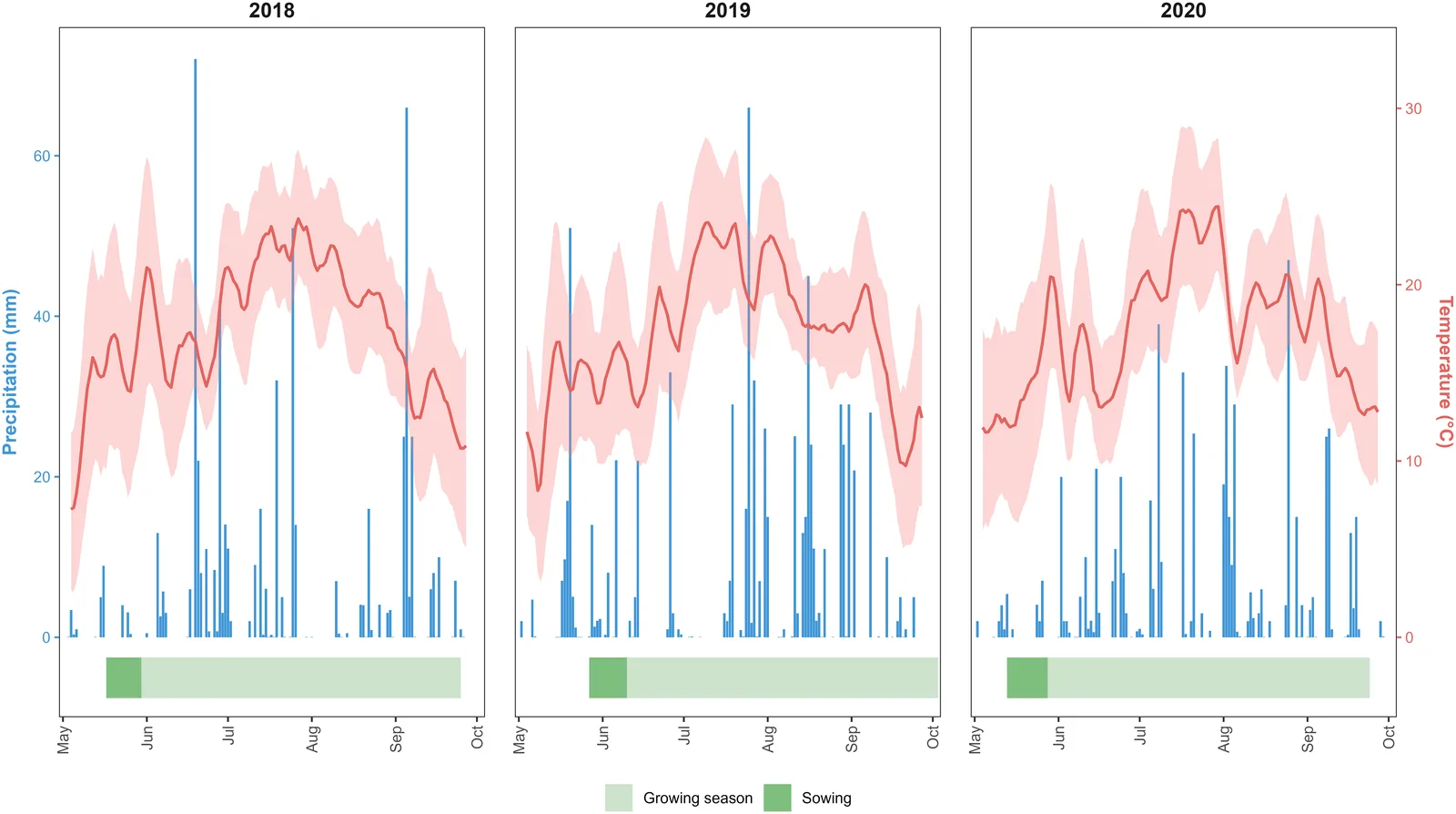
Weather during the three growing seasons (2018–2020) at the FERIA field station, Khabarovsk Krai, Russia. Bars show daily precipitation. The line shows the 7-day rolling mean air temperature and the band shows the range between daily minimum and maximum temperatures.

Field experiments were conducted over three consecutive growing seasons (2018–2020) at the experimental field station of the Far Eastern Research Institute of Agriculture (FERIA), Vostochnoye village (48°N, 135°E), Khabarovsk Krai, Russia. The site lies within a humid continental climate zone (**Figure 1**). Daily air temperature and precipitation records for the Khabarovsk meteorological station were obtained from the Reliable Prognosis archive (rp5.ru). Mean air temperature over the growing season was similar across the three years (17.8–18.1 °C), as was total precipitation (566–631 mm). The within-season distribution, however, differed markedly. August precipitation ranged from 44 mm in 2018 to 251 mm in 2019, and June precipitation from 98 mm in 2019 to 213 mm in 2018, whilst mean May temperature ranged from 12.9 to 16.3 °C. Monthly values and the agrochemical properties of the experimental soil are given in **Supplementary Tables 1** and **2**.

A panel of 63 soybean (*Glycine max* (L.) Merr.) accessions of diverse geographical origin was evaluated; the name and country of origin of each accession are listed in **Supplementary Table 3**. Each season, the experiment was arranged in a randomised complete block design with two to three replications, and the same set of genotypes was grown in all three years. Crop management followed standard regional practice and was applied uniformly across seasons. Pre-sowing fertilisation was applied at 40 kg ha^-^¹ each of nitrogen, phosphorus, and potassium on an active-ingredient basis. Sowing was carried out in mid-to-late May on raised beds at a row spacing of 70 cm and an in-row spacing of 10 cm, corresponding to a density of approximately 14.3 plants m^-^². Weeds were controlled by pre- and post-emergence herbicides, mechanical inter-row cultivation, and manual weeding. Seed was not inoculated, and no fungicides or insecticides were applied. The trials were rainfed throughout. Plants were harvested manually at technical maturity. Plant height (cm), first pod height (cm), number of branches, number of productive nodes on the main stem, and number of productive nodes on the lateral branches were recorded on representative plants of each accession. Days to maturity was recorded as the number of days from sowing to technical maturity.

### 2.2 Statistical analysis

#### 2.2.1 Analysis of variance

All statistical analyses were performed in R (version 4.4.0; R Core Team, 2024). Descriptive statistics were computed for each trait across all observations, including the mean, standard deviation, minimum, maximum, skewness, kurtosis, and the coefficient of variation (CV, expressed as the standard deviation as a percentage of the mean). A combined analysis of variance was performed across the three years to partition the phenotypic variation in each trait. Because the genotypes were evaluated over consecutive years at a single site, each year was treated as a distinct environment. The following linear model in Equation 1 was fitted for each trait:

(1) $$Y_{ijk}=\mu +G_{i}+E_{j}+{\left(GE\right)}_{ij}+R_{k\left(j\right)}+\epsilon_{ijk}$$

where *Y_ijk_* is the observed phenotypic value of the i-th genotype in the k-th replication of the j-th year; *μ* is the overall mean; *G_i_* is the effect of the i-th genotype; *E*_j_ is the effect of the j-th year (environment); (*GE*)*_ij_* is the genotype × year interaction; *R_k_*_(_*_j_*_)_ is the effect of the k-th replication nested within the j-th year; and *ϵ_ijk_* is the residual error. Because the replication was unbalanced (two to three replicates per genotype–year combination) and the genotype × year interaction was significant for every trait, Type III sums of squares were used, computed with sum-to-zero contrasts (Fox and Weisberg, 2019), so that each term was tested after adjustment for all others. Model assumptions were evaluated for each trait. Normality of the residuals was assessed by the Shapiro-Wilk test, together with residual skewness and kurtosis and quantile-quantile plots, and homogeneity of variance across years by Levene’s test and residual-versus-fitted plots. Because formal tests are highly sensitive to minor departures at this number of observations, the diagnostic plots were given primary weight, and the analysis was considered robust to the mild departures observed.

Year-to-year variation in each trait was characterised by estimated marginal means obtained from a model including year and genotype effects, with pairwise differences between years tested using Tukey’s adjustment for multiple comparisons (Lenth, 2024).

#### 2.2.2 Estimation of variance components and genetic parameters

To estimate the variance components and genetic parameters underlying each trait, the model of Equation 1 was refitted as a linear mixed model with all effects treated as random and assumed to be normally and independently distributed as *G_i_ ~ N(0, σ²g), E_j_ ~ N(0, σ²e), GE_ij_ ~ N(0, σ²ge), R_k(j)_ ~ N(0, σ²r), and ϵ_ijk_ ~ N(0, σ²)* for the genotype, year, genotype by year, replication within year, and residual terms, respectively. Treating genotypes as a random sample allows the phenotypic variance to be partitioned into genetic and non-genetic components (Piepho et al., 2008). Best linear unbiased predictions (BLUPs) of the genotypic effects, that is, predicted genetic values expressed as deviations from the overall mean, were obtained for the 63 genotypes. Because a variance component estimated from three years is imprecise, the models were also refitted with year as a fixed effect; broad-sense heritabilities and genotypic BLUPs were unchanged (*r* = 1.000). The predictive ability of the genotypic BLUPs was assessed by leave-one-year-out cross-validation. For each year in turn, the model was refitted on the remaining two years and the resulting BLUPs were correlated with the observed genotype means in the held-out year.

The significance of the genotypic variance component was assessed by a likelihood ratio test (LRT), comparing the full model with a reduced model from which the genotype term had been removed (Equation 2):

(2) $$LRT=-2\left(logL_{0}-logL_{1}\right)$$

where *L*_0_ and *L*_1_ are the likelihoods under the null (no genotypic effect) and full specifications. The test statistic was referenced to a chi-square distribution with one degree of freedom.

The phenotypic variance on a genotype-mean basis was computed as shown in Equation 3:

(3) $$\sigma_{p}^{2}=\sigma_{g}^{2}+\frac{\sigma_{ge}^{2}}{y}+\frac{\sigma^{2}}{yr}$$

where y is the number of years (3) and r is the harmonic mean of the number of replications per genotype-year combination, which accounts for the unbalanced replication. Broad-sense heritability on a genotype-mean basis (Holland et al., 2003), the genotypic and phenotypic coefficients of variation as percentages of the trait mean, and the expected genetic advance under selection were then estimated as shown in Equations 4–7, respectively:

(4) $$H^{2}=\frac{\sigma_{g}^{2}}{\sigma_{p}^{2}}$$

(5) $$GCV\left(\%\right)=\frac{\sqrt{\sigma_{g}^{2}}}{\bar{x}}\times 100$$

(6) $$PCV\left(\%\right)=\frac{\sqrt{\sigma_{p}^{2}}}{\bar{x}}\times 100$$

(7) $$GA=k\times \sqrt{\sigma_{p}^{2}}\times H^{2}$$

where *k* is the standardised selection differential, taken as 2.06 for a selection intensity of 5%. Genetic advance was also expressed as a percentage of the mean, GAM (%) = (GA/x¯) × 100, and selective accuracy was estimated as the square root of the broad-sense heritability. Mixed models were fitted using the lme4 package (Bates et al., 2015), with likelihood ratio tests for random effects performed using lmerTest (Kuznetsova et al., 2017). Figures were produced using ggplot2 (Wickham, 2016).

### 2.3 Trait interrelationships and multivariate structure

Phenotypic correlations amongst the six traits were computed as Pearson correlations amongst genotype means across the three years, and genotypic-value correlations as Pearson correlations amongst the genotypic BLUPs obtained in section 2.2.2. The multivariate structure of architectural variation was characterised by principal component analysis of the genotypic values, based on the correlation matrix to account for differences in trait scale. The number of informative components was assessed from the proportion of variance explained, and the axes were interpreted from the trait loadings. Genotypes were grouped by hierarchical cluster analysis of the Euclidean distances amongst their standardised genotypic values, using Ward’s minimum-variance method (Ward.D2) (Ward, 1963; Murtagh and Legendre, 2014). The number of clusters was assessed using the silhouette (Rousseeuw, 1987) and gap-statistic (Tibshirani et al., 2001) criteria together with inspection of the dendrogram. Analyses were conducted in R using the stats, factoextra (Kassambara and Mundt, 2020), cluster (Maechler et al., 2023), and GGally (Schloerke et al., 2024) packages.

### 2.4 Multi-trait genotype-ideotype distance index

Genotype selection towards a defined plant-architecture ideotype was performed using the multi-trait genotype-ideotype distance index (MGIDI) (Olivoto and Nardino, 2021). The ideotype was defined by the direction of change sought in each trait rather than by absolute target values, following regional breeding priorities for the Russian Far East. Earlier maturity was sought because the frost-free period limits the length of the growing season. Taller plants and a higher first pod were sought because lodging was assumed not to constrain plant height at the moderate densities used in the region, and because pods borne below the cutter bar are lost at harvest. More branches and more productive nodes were sought because they increase the number of pod-bearing sites. Days to maturity was therefore assigned a desired direction of decrease and the five architectural traits a desired direction of increase. MGIDI was computed from the genotypic values (BLUPs), following a factor analysis in which factors with eigenvalues greater than one (Kaiser criterion) (Kaiser, 1960) were retained and rotated by varimax. For each genotype, the index was calculated as the Euclidean distance between its factor scores and those of the ideotype (Equation 8):

(8) $$MGIDI_{i}=\sqrt{\sum_{j}{\left(F_{ij}-F_{j}\right)}^{2}}$$

where *F*_ij_ is the score of the i-th genotype on the j-th factor, and *F*_j_ is the ideotype score on that factor. The genotype with the lowest MGIDI value is closest to the ideotype.

Because the size traits were positively correlated with later maturity, a range of size-trait weightings (0.5 to 1.0) was explored, with full weight retained on days to maturity (**Supplementary Table 4**). At a weighting of 0.5, plant height and first pod height responded in the undesired direction, whereas at weightings of 0.8 or above days to maturity increased. A weighting of 0.6 was therefore adopted, this being the lowest weighting at which plant height and first pod height improved whilst days to maturity still decreased. A selection intensity of 15% was applied. Analyses were performed in R using the metan package (Olivoto and Lúcio, 2020).

### 2.5 Multi-trait stability index

Genotypes combining proximity to the ideotype with stability across years were identified using the multi-trait stability index (MTSI) (Olivoto et al., 2019b). Genotypic stability for each trait was first quantified by the weighted average of absolute scores (WAASB) from a linear mixed model with genotype and the genotype by year interaction as random effects and year as fixed, treating years as environments. The WAASB values were then combined with trait means to give WAASBY values, which integrate mean performance and stability. MTSI was computed on those values using the same desired directions as for MGIDI, with equal weighting across traits. Factors with eigenvalues greater than one were retained and rotated by varimax, and a selection intensity of 15% was applied. Analyses were performed in R using the metan package (Olivoto and Lúcio, 2020).

## 3 Results

### 3.1 Phenotypic variation and analysis of variance

The six traits showed a wide range of variation across the 63 genotypes (**Table 1**). Mean plant height was 63.5 cm (21.8 to 127.6 cm) and mean first pod height 9.7 cm (1.9 to 27.4 cm). Genotypes averaged 2.9 branches (0 to 10.2), 10.8 productive nodes on the main stem (2.6 to 19.0), and 12.0 productive nodes on lateral branches (0 to 39.6). Days to maturity averaged 131.4 days and ranged from 104 to 159 days. The coefficient of variation was highest for the number of productive nodes on lateral branches (67.9%) and the number of branches (64.4%), intermediate for first pod height and plant height (45.3% and 28.3%), and lowest for the number of productive nodes on the main stem (22.3%) and days to maturity (9.4%). The branching traits therefore showed the highest variability amongst genotypes, whereas the phenological and main-stem traits showed low variability.

**Table 1:** Descriptive statistics for the six traits in 63 soybean genotypes, 2018–2020 (*n* = 492).

Trait	Mean	SD	Min	Max	Skewness	Kurtosis	CV (%)
Plant height	63.47	17.94	21.8	127.6	0.49	0.33	28.26
First pod height	9.74	4.41	1.9	27.4	0.75	0.38	45.26
Number of branches	2.89	1.86	0	10.2	0.80	0.71	64.37
Productive nodes on main stem	10.80	2.41	2.6	19.0	-0.10	0.18	22.32
Productive nodes on lateral branches	11.96	8.12	0	39.6	0.80	0.38	67.91
Days to maturity	131.36	12.30	104	159	0.56	-0.40	9.36

SD, standard deviation; CV, coefficient of variation.

The combined analysis of variance revealed highly significant effects of genotype, year, and the genotype by year interaction for all six traits (*p* < 0.001; **Table 2**). The genotype effect indicates significant genetic variation amongst the 63 genotypes, and the year effect indicates environmental variation across the three growing seasons. The genotype by year interaction, significant for every trait, indicates differential genotype responses amongst years. The replication effect nested within year was non-significant for all traits except the number of productive nodes on the main stem, indicating generally homogeneous conditions within years. Quantile-quantile and residual-versus-fitted plots for each trait are given in **Supplementary Figures 1**, **2**.

**Table 2:** Type III mean squares from the combined analysis of variance for the six traits in 63 soybean genotypes, 2018–2020.

Trait	Year	Replication (year)	Genotype	Genotype × year
Plant height	19970.37***	46.41	1159.56***	135.39***
First pod height	1508.53***	7.69	41.80***	14.70***
Number of branches	122.73***	0.93	11.24***	2.65***
Productive nodes on main stem	49.63***	6.31*	23.13***	4.81***
Productive nodes on lateral branches	1366.26***	16.49	243.71***	53.56***
Days to maturity	1088.19***	36.94	867.52***	75.76***

df: Year = 2, Replication (Year) = 6, Genotype = 62, Genotype × Year = 124, Residual = 297. ***p< 0.001, *p< 0.05.

**Figure 2:**
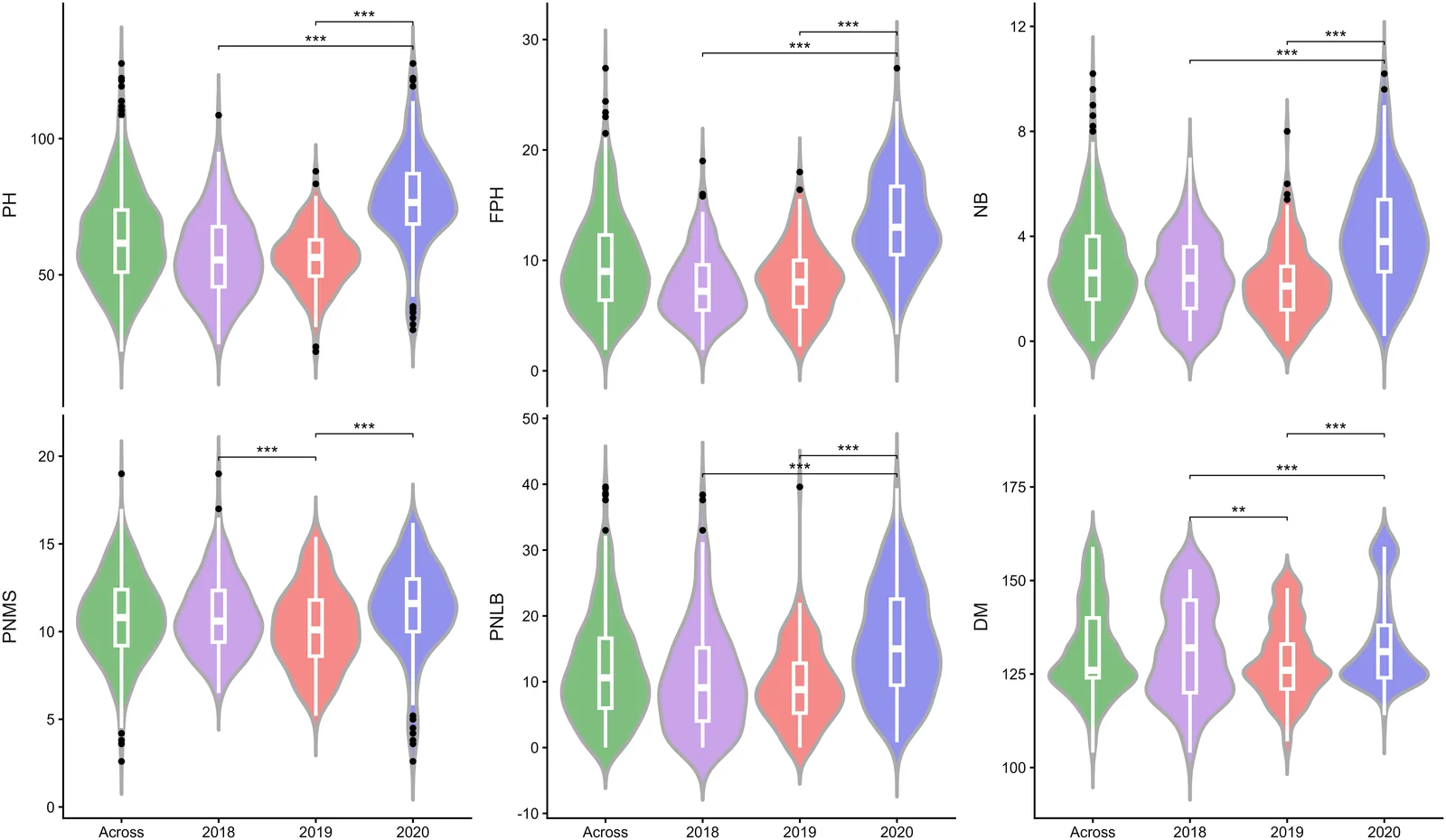
Distribution of the six traits in each of the three years and pooled across years (“Across”), shown as violin plots with embedded box plots. Brackets denote significant pairwise comparisons between years from Tukey-adjusted estimated marginal means; ***p < 0.001, **p< 0.01, *p < 0.05. Trait abbreviations: PH, plant height; FPH, first pod height; NB, number of branches; PNMS, productive nodes on main stem; PNLB, productive nodes on lateral branches; DM, days to maturity.

Pairwise comparisons of estimated marginal means (**Figure 2**) showed that 2020 differed significantly from the other two years for plant height, first pod height, number of branches, and productive nodes on lateral branches, whereas 2018 and 2019 did not differ for these traits. Days to maturity differed significantly amongst all three years, and the number of productive nodes on the main stem followed a different pattern, with 2019 differing from both other years.

### 3.2 High heritability across traits, with the greatest genetic advance in the branching traits

**Figure 3:**
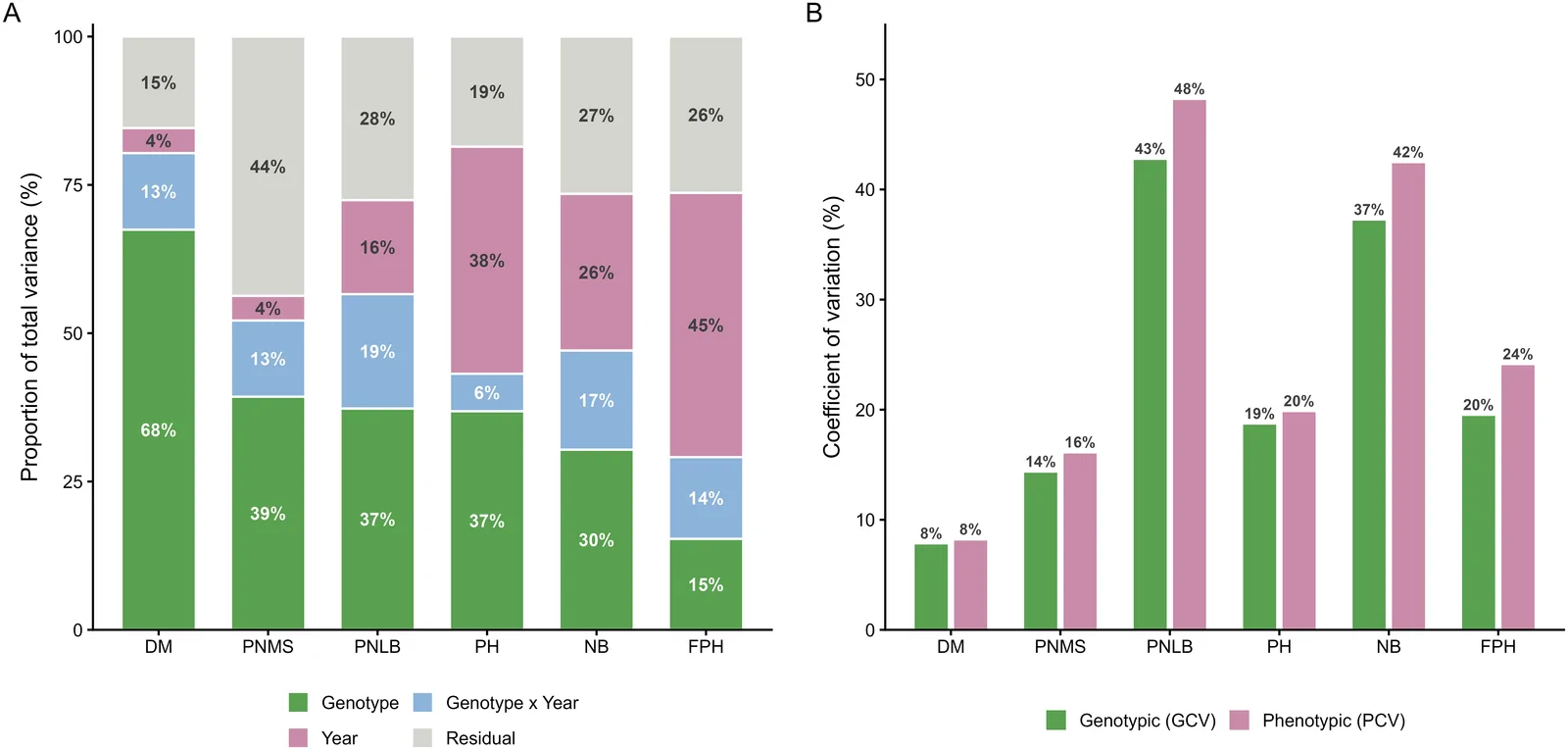
Genetic parameters for the six traits. **(A)** Partitioning of the phenotypic variance into genotype, year, genotype by year, and residual components, as a percentage of the total, ordered by genotypic share. **(B)** Genotypic (GCV) and phenotypic (PCV) coefficients of variation. Trait abbreviations are defined in **Figure 2**.

Partitioning of the phenotypic variance showed that the genotypic component was predominant for days to maturity, the year component was substantial for plant height and first pod height, and the residual component was largest for productive nodes on the main stem (**Figure 3**). The genotypic variance was highly significant for all six traits (likelihood ratio test, *p<* 0.001; **Supplementary Table 5**).

Broad-sense heritability was moderate to high across all traits, ranging from 0.65 for first pod height to 0.91 for days to maturity (**Table 3**). Days to maturity (0.91), plant height (0.89), and productive nodes on the main stem (0.80) showed higher heritability values, indicating the predominance of genetic control for these traits. For every trait the phenotypic coefficient of variation only slightly exceeded the genotypic coefficient of variation, the two differing by 0.4 to 5.4 percentage points (**Table 3**), indicating that the observed phenotypic variation was largely genetic in origin and subject to limited environmental influence. The genotypic coefficient of variation was higher for productive nodes on lateral branches (42.8%) and number of branches (37.3%) and lowest for days to maturity (7.8%), reflecting higher genetic variability in the branching traits.

**Table 3:** Genetic parameters for the six traits in 63 soybean genotypes, 2018–2020.

Trait	Mean	*H²*	GCV (%)	PCV (%)	GA	GAM (%)	Accuracy
PH	63.47	0.89	18.73	19.86	23.10	36.39	0.94
FPH	9.74	0.65	19.52	24.13	3.17	32.53	0.81
NB	2.89	0.77	37.26	42.47	1.95	67.33	0.88
PNMS	10.80	0.80	14.37	16.11	2.85	26.40	0.89
PNLB	11.96	0.79	42.78	48.22	9.35	78.17	0.89
DM	131.36	0.91	7.84	8.20	20.29	15.44	0.96

H², broad-sense heritability; GCV and PCV, genotypic and phenotypic coefficients of variation; GA, genetic advance; GAM, genetic advance as a percentage of the mean; Accuracy, selective accuracy (√H²). Trait abbreviations are defined in **Figure 2**.

**Figure 4:**
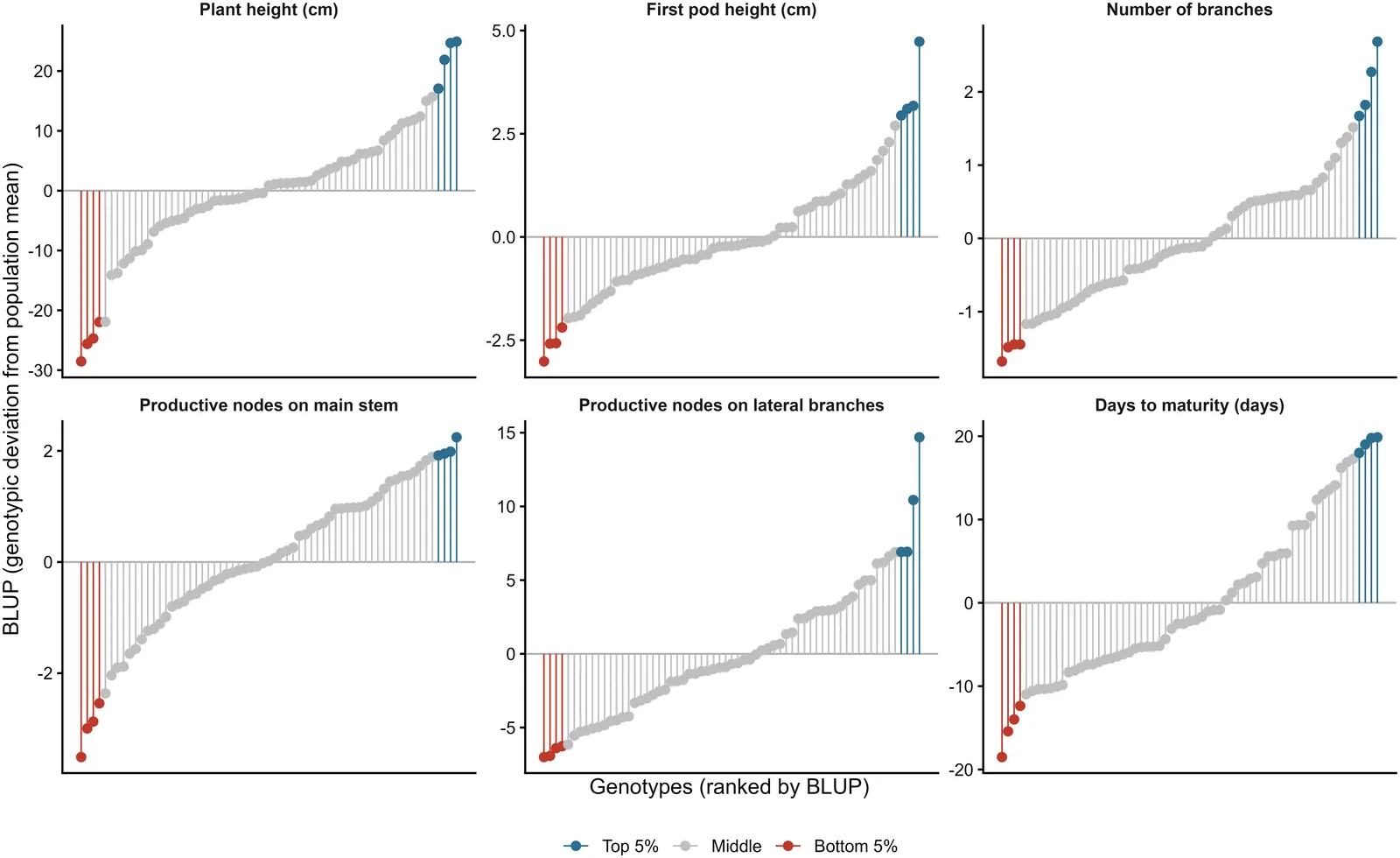
Genotypic BLUPs for the six traits, with genotypes ordered by BLUP value within each panel. The top and bottom 5% of genotypes are highlighted in blue and red, respectively, and the horizontal line marks the population mean.

Genetic advance as a percentage of the mean ranged widely amongst traits, from 15.4% for days to maturity to 78.2% for productive nodes on lateral branches (**Table 3**). Productive nodes on lateral branches (78.2%) and number of branches (67.3%) combined high heritability with high genetic advance. Days to maturity showed the highest heritability but the lowest genetic advance, having the lowest phenotypic variation (PCV 8.2%, against 42.5% and 48.2% for the two branching traits). The accuracy of selection was high for all traits (0.81 to 0.96). Leave-one-year-out cross-validation gave predictive abilities of 0.48 to 0.84 (**Supplementary Table 5**), in the same rank order as the heritabilities. Dividing by the square root of the heritability of a single-year genotype mean gives predictive accuracies of 0.77 to 0.96, which agree with the selective accuracies in **Table 3** to within 0.04 for every trait. The relationship between heritability and genetic advance is shown in **Supplementary Figure 3**. The distribution of BLUP values was symmetrical around the population mean for each trait, with a small number of genotypes at the extremes showing the highest and lowest genotypic values (**Figure 4**). The genotypic BLUPs for all 63 genotypes are given in **Supplementary Table 6**.

### 3.3 Architectural variation is structured along two largely independent dimensions

**Figure 5:**
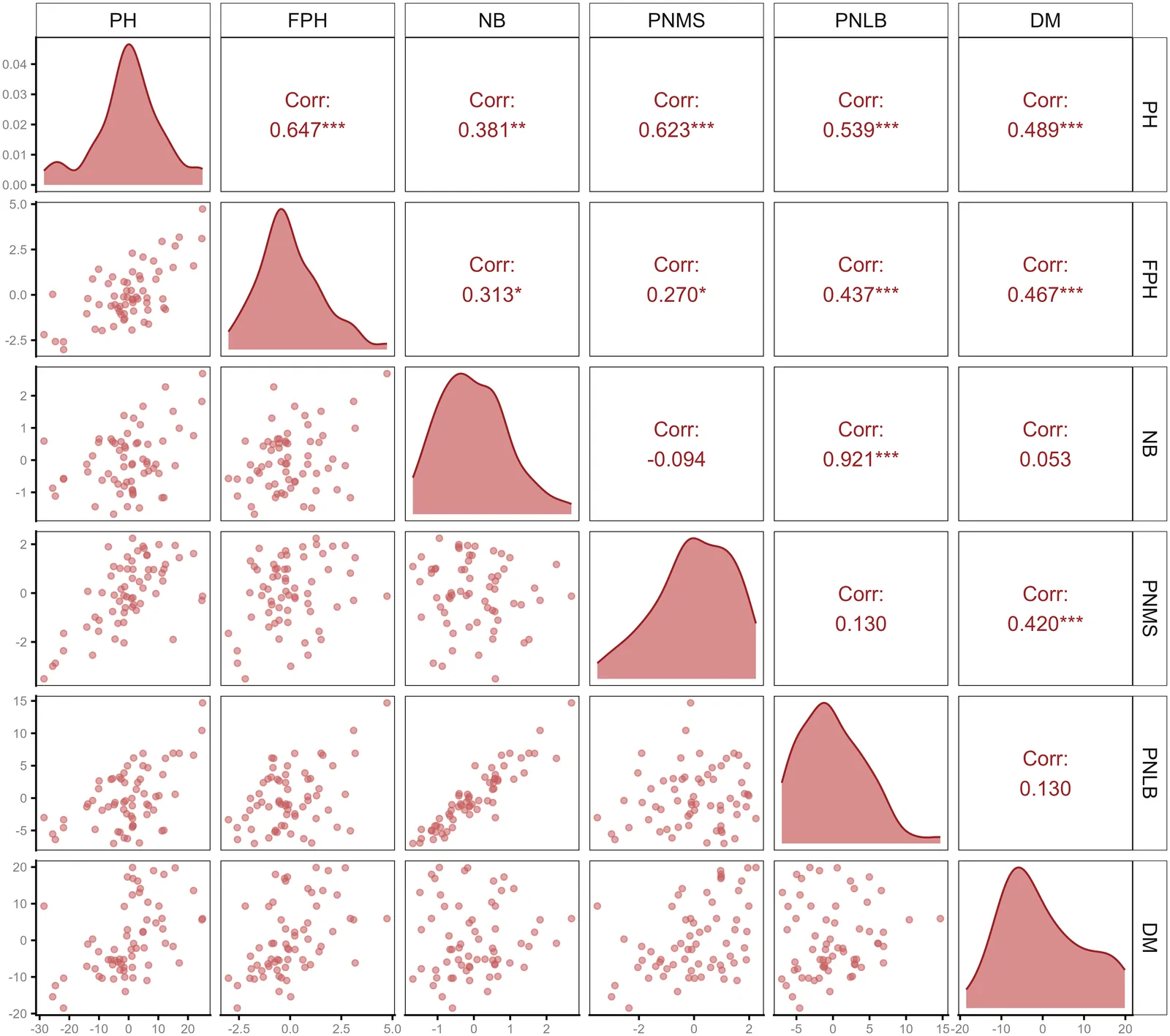
Distributions and pairwise relationships of genotypic values for the six traits. Diagonal panels, density distributions; lower triangle, pairwise scatterplots; upper triangle, Pearson correlation coefficients. ***p < 0.001, **p < 0.01, *p < 0.05. Trait abbreviations are defined in **Figure 2**.

Correlations amongst the genotypic values are shown in **Figure 5**. The strongest was between the number of branches and the number of productive nodes on lateral branches (*r* = 0.92). Plant height was moderately and positively correlated with every other trait (*r* = 0.38 to 0.65). The number of productive nodes on the main stem, by contrast, showed no significant correlation with the branching traits (*r* = -0.09 to 0.13), so branching and main-stem node number varied largely independently amongst genotypes. Days to maturity was moderately correlated with the main-axis traits but only weakly with the branching traits.

**Figure 6:**
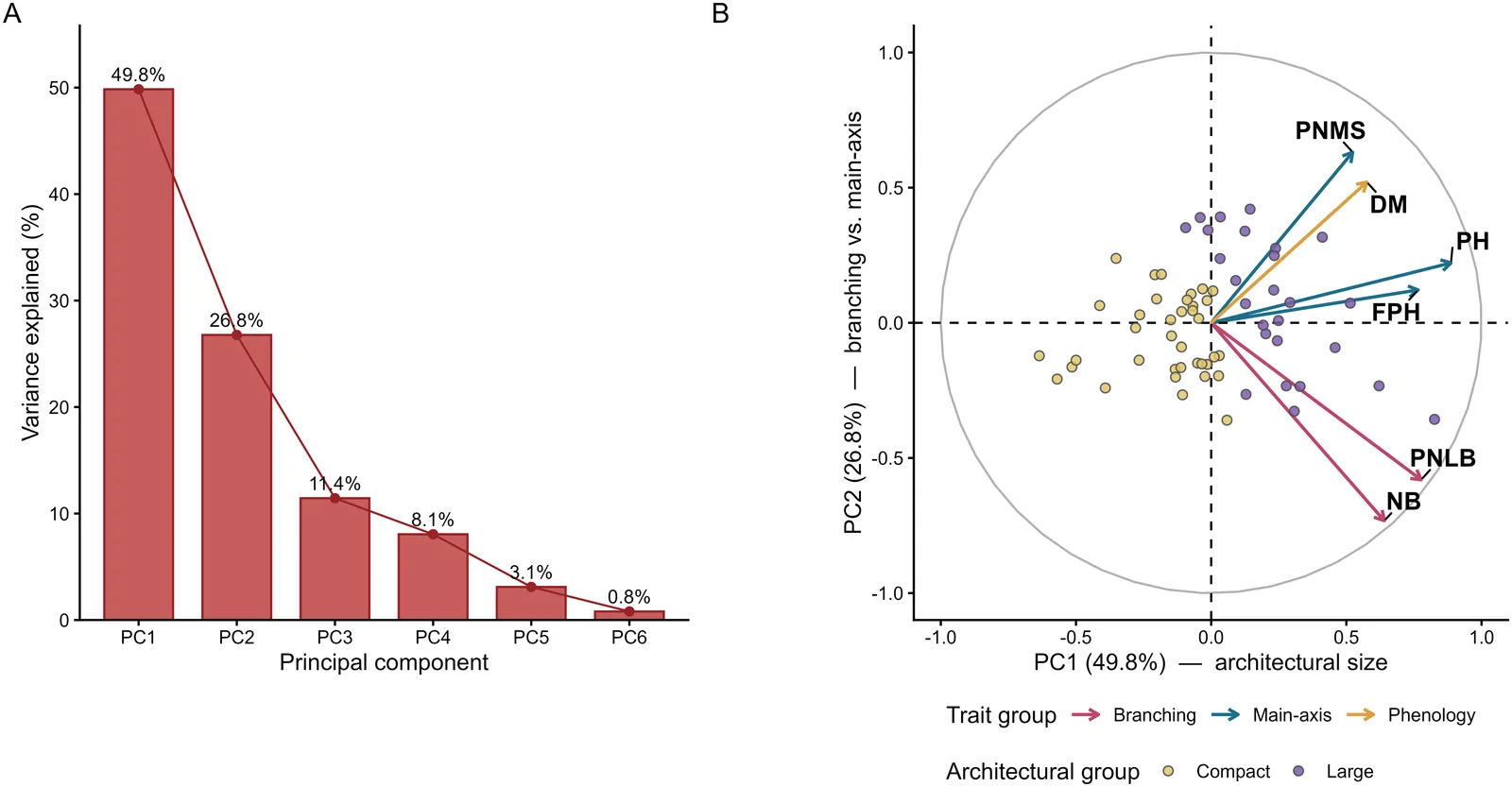
Principal component analysis and hierarchical clustering of genotypic values for the six traits. **(A)** Proportion of variance explained by each principal component. **(B)** Biplot of the first two components. Trait vectors are coloured by architectural group (branching, main-axis, and phenology) and genotypes by hierarchical cluster. PC1 (49.8%) reflects overall architectural size and PC2 (26.8%) contrasts branching traits with main-axis and phenological traits. Trait abbreviations are defined in **Figure 2**.

The phenotypic-genotypic correlation coefficients were similar in both magnitude and direction across all trait pairs (**Figure 5**; **Supplementary Table 7**). Principal component analysis of the genotypic values reduced this correlation structure to a small number of independent axes (**Figure 6**). The first two principal components together accounted for 76.6% of the total variation (PC1 = 49.8%, PC2 = 26.8%), with the remaining components accounting for progressively smaller proportions. All six traits loaded positively on the first component, indicating that PC1 represented an axis of overall architectural size, separating genotypes that were uniformly larger in stature, branching, and node number from those that were smaller.

The second component contrasted the branching traits, which loaded negatively, with the main-stem and phenological traits, which loaded positively. This axis therefore distinguished genotypes with more branches and lateral-branch nodes from those with more main-stem nodes and later maturity, consistent with the near-zero correlation between these trait groups.

Hierarchical clustering of the genotypic values divided the panel into two architectural groups that separated primarily along the first principal component (**Figure 6B**). One comprised more compact, earlier-maturing genotypes (mean plant height 56.5 cm, 124.8 days to maturity), the other larger, later-maturing genotypes with more nodes and branches (mean plant height 73.3 cm, 141.1 days to maturity). Neither validation criterion identified a distinct optimum: the average silhouette width was almost identical for two, three and four clusters (0.272, 0.272 and 0.274), and the gap statistic did not support subdivision (**Supplementary Figure 4**). Two clusters were adopted from the dendrogram structure, and the resulting groups were partly overlapping, consistent with the largely continuous architectural variation observed in the ordination.

### 3.4 MGIDI identifies the genotypes closest to the ideotype

**Figure 7:**
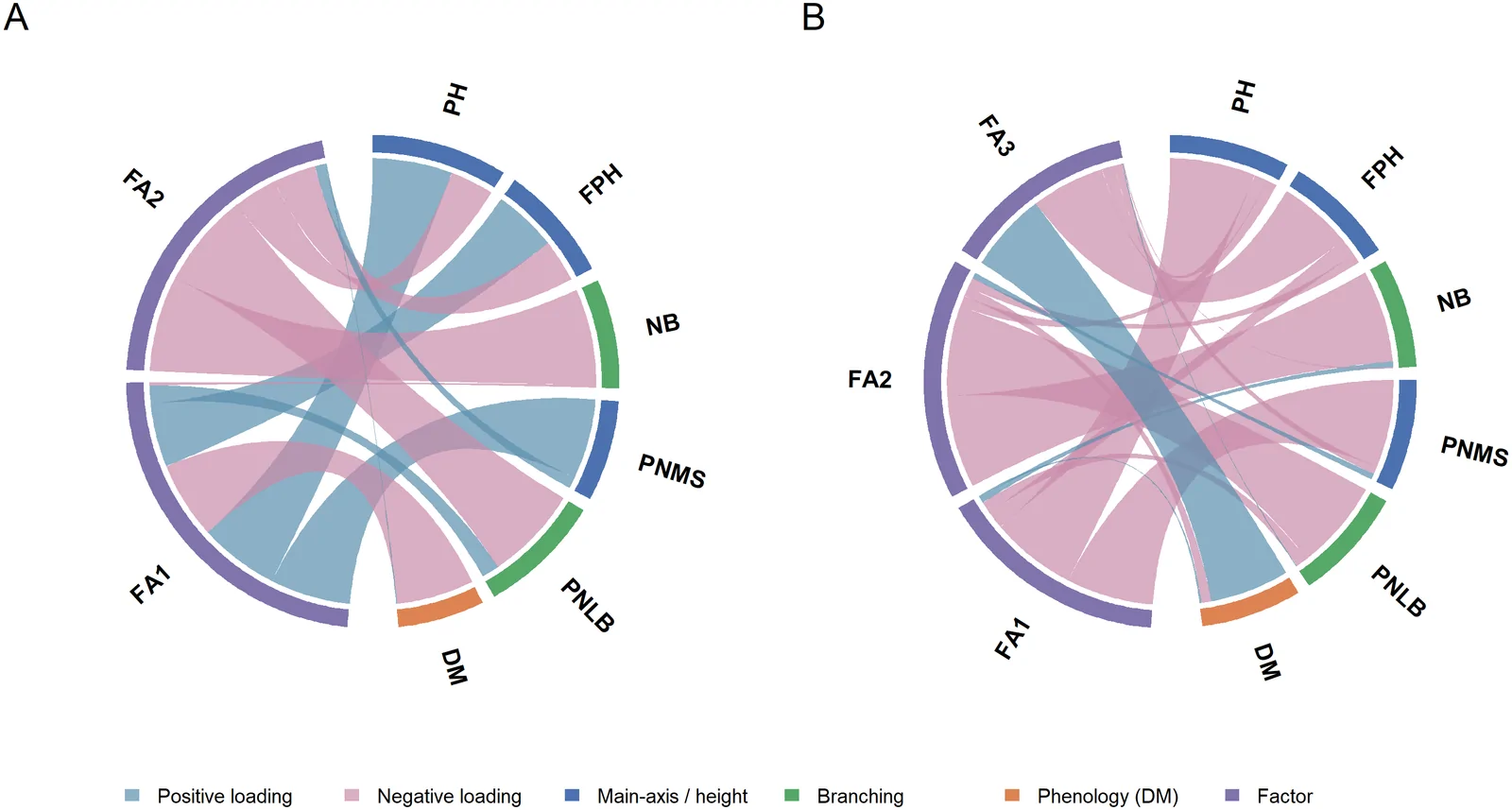
Trait-to-factor loading structure from the factor analyses underlying the two selection indices. **(A)** MGIDI, which retained two factors, and **(B)** MTSI, which retained three. Ribbon width is proportional to the absolute loading. Trait abbreviations are defined in **Figure 2**.

The factor analysis underlying MGIDI retained two factors (eigenvalues > 1) that together explained 76.5% of the variation, consistent with the structure identified by the principal component analysis (**Table 4**). The first factor was defined by plant height, first pod height, productive nodes on the main stem, and days to maturity. Maturity loaded in the opposite direction to the three size traits, so the factor represents a size and maturity dimension. The second factor was defined by the two branching traits alone (loadings -0.97 and -0.95) and represents an independent branching dimension (**Figure 7A**). Communalities were moderate to high (0.60 to 0.95), indicating that the two factors accounted for most of the variation in all six traits.

**Table 4:** Factor loadings, communalities, and uniquenesses for the six traits from the factor analysis underlying MGIDI.

Trait	FA1	FA2	Communality	Uniqueness
PH	-0.80	-0.44	0.84	0.16
FPH	-0.64	-0.43	0.60	0.40
NB	0.03	-0.97	0.95	0.05
PNMS	-0.81	0.11	0.67	0.33
PNLB	-0.18	-0.95	0.94	0.06
DM	0.78	0.01	0.60	0.40

Trait abbreviations are defined in **Figure 2**.

FA1 and FA2 are the two retained factors (eigenvalues > 1).

**Figure 8:**
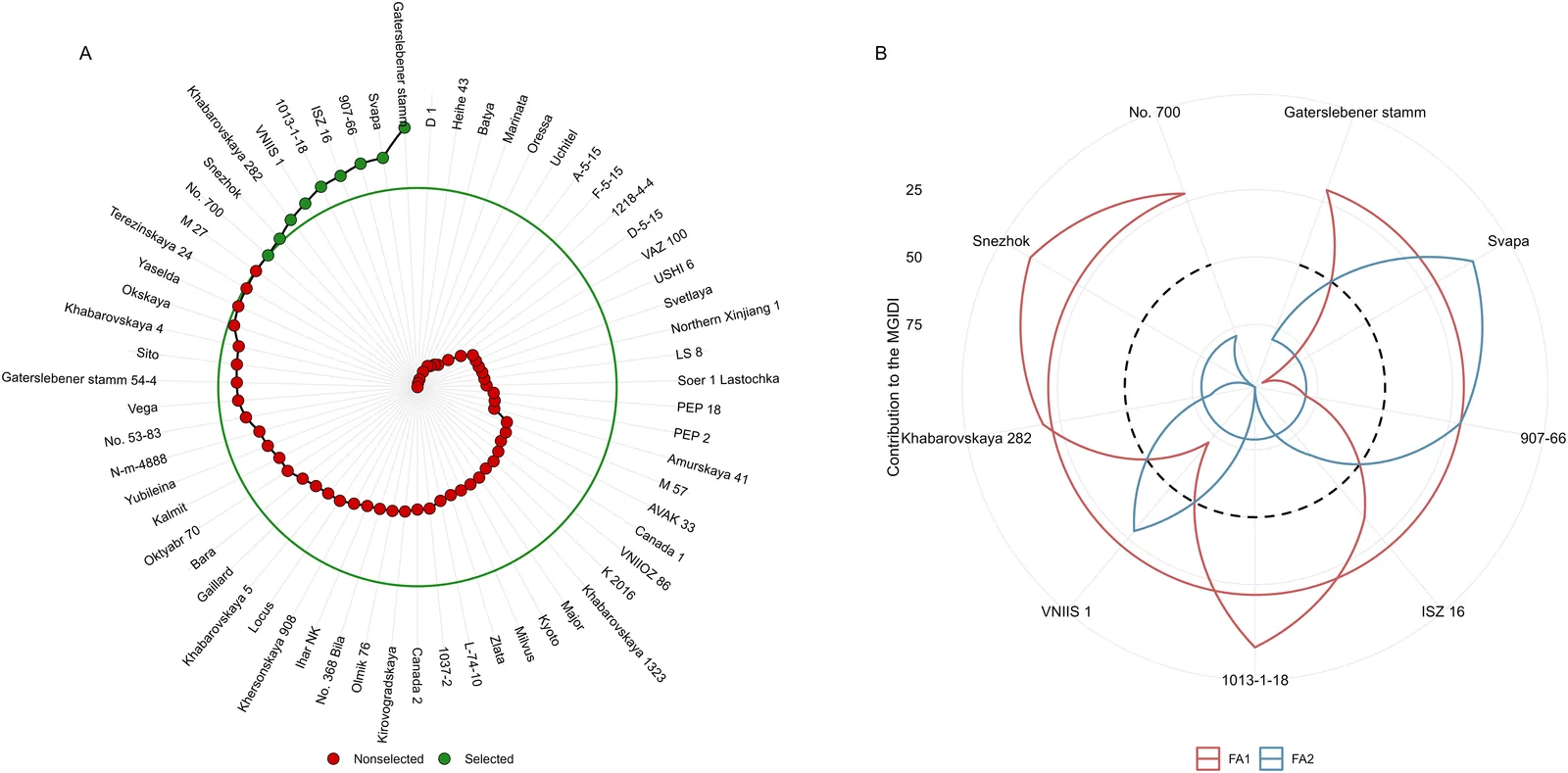
Genotype ranking by the multi-trait genotype-ideotype distance index (MGIDI). **(A)** Genotypes are ordered from the centre outward by increasing MGIDI value; those within the cut-off circle were selected at 15% selection intensity (green) and those outside were not (red). **(B)** Contribution of each factor (FA1, FA2) to the MGIDI value of the selected genotypes; the dashed line is the value expected if all factors contributed equally.

At 15% selection intensity, nine genotypes were selected as closest to the ideotype (**Figure 8**). These were Gaterslebener stamm, Svapa, 907-66, ISZ 16, 1013-1-18, VNIIS 1, Khabarovskaya 282, Snezhok, and No. 700. The selection differentials were in the desired direction for five of the six traits (**Table 5**). Plant height and first pod height increased by 2.9% and 2.3% and days to maturity decreased by 2.3%, so the selected genotypes combined a larger, higher-podded architecture with earlier maturity. The branching traits showed the highest selection differentials, the number of branches increasing by 39.9% and productive nodes on lateral branches by 36.1%. The number of productive nodes on the main stem was the only trait to move against its desired direction, decreasing by 6.1% due to its genetic association with the later maturity that selection acted against.

**Table 5:** Selection differentials for the six traits at 15% selection intensity under MGIDI. Traits are grouped by the factor on which they loaded.

Trait	Factor	Xo	Xs	SD%	Desired
PH	FA1	63.47	65.32	2.9	↑
FPH	FA1	9.74	9.96	2.3	↑
PNMS	FA1	10.80	10.14	-6.1	↑
DM	FA1	131.36	128.32	-2.3	↓
NB	FA2	2.89	4.04	39.9	↑
PNLB	FA2	11.96	16.28	36.1	↑

Xo, mean of all genotypes; Xs, mean of selected genotypes; SD%, selection differential as a percentage of the mean; Desired, desired direction of selection (↑ increase, ↓ decrease). Trait abbreviations are defined in **Figure 2**.

### 3.5 Multi-trait selection for stability complements ideotype selection

The factor analysis underlying MTSI retained three factors with eigenvalues greater than one, which together explained 77.0% of the variation (**Figure 7B**; **Supplementary Table 8**). The first factor grouped plant height and productive nodes on the main stem, the second the two branching traits, and the third first pod height and days to maturity. At 15% selection intensity, MTSI selected nine genotypes. These were Gaterslebener stamm, Amurskaya 41, Kalmit, VNIIS 1, ISZ 16, Zlata, Yaselda, Canada 2, and No. 700. In contrast to MGIDI, all six selection differentials under MTSI were in the desired direction (**Supplementary Table 9**), including that for productive nodes on the main stem (+1.3%), which had decreased under MGIDI selection. The highest selection differentials were again in the branching traits, with the number of branches increasing by 44.1% and productive nodes on lateral branches by 48.0%, whilst plant height increased by 14.8%, first pod height by 9.4%, and days to maturity decreased by 1.9%.

**Figure 9:**
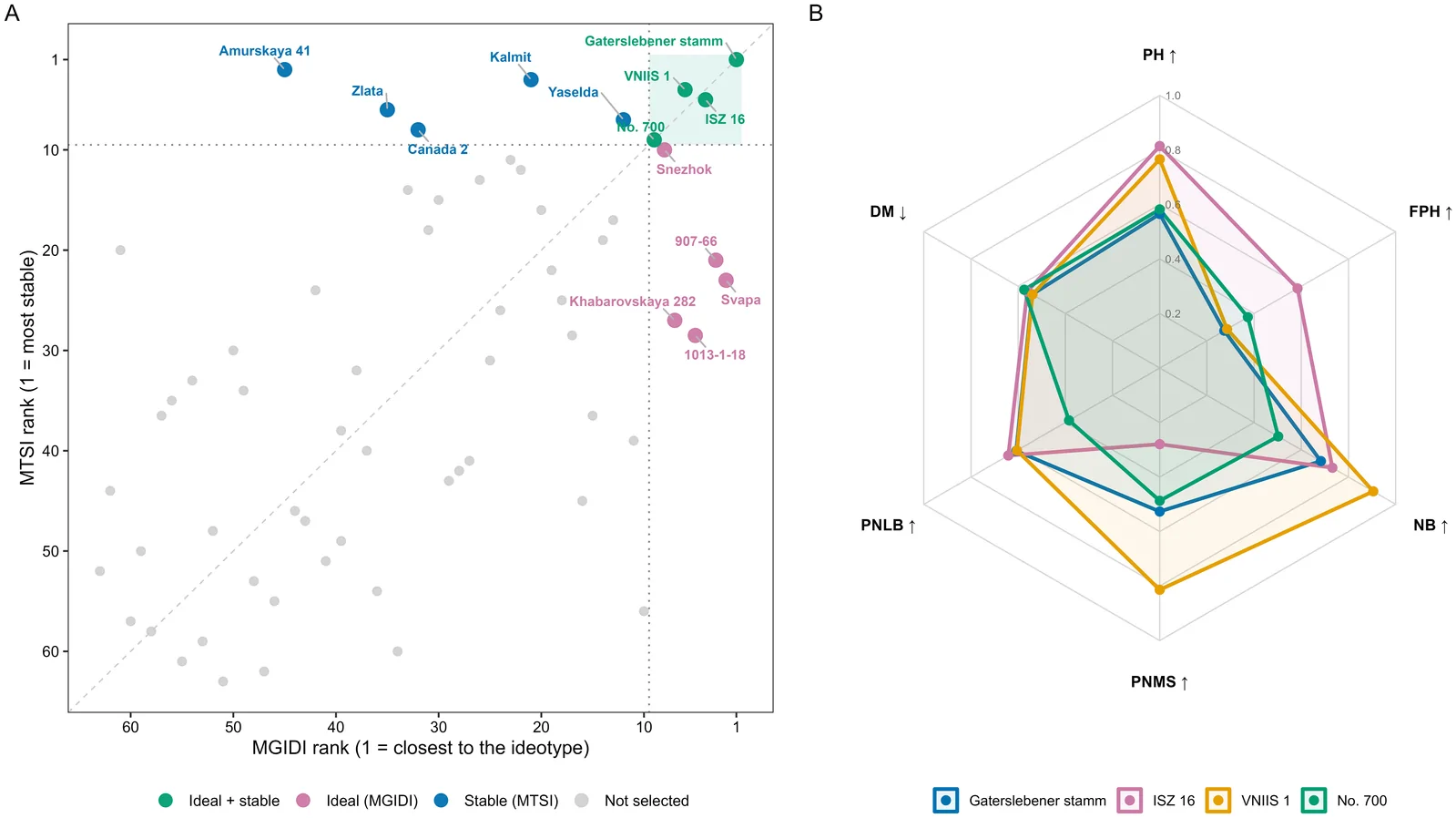
Multi-trait selection of the 63 genotypes. **(A)** MGIDI rank against MTSI rank; axes are reversed so that the more desirable direction is up and to the right. Dotted lines mark the 15% selection thresholds and the dashed diagonal equal rank on the two indices. **(B)** Trait profiles of the four genotypes selected by both indices, scaled from 0 to 1 across all 63 genotypes and oriented so that outer values are more favourable; arrows give the desired direction (↑ higher, ↓ lower). Trait abbreviations are defined in **Figure 2**.

Four genotypes, Gaterslebener stamm, VNIIS 1, ISZ 16, and No. 700, were selected by both indices (**Figure 9A**), and these combine proximity to the architectural ideotype with temporal stability. Gaterslebener stamm ranked first under both indices. Five genotypes were selected by MGIDI only, which are close to the ideotype but less temporally stable, and five by MTSI only, which are stable but more distant from the ideotype. The four genotypes selected by both indices are therefore the most promising candidates, combining the target architecture with consistent performance across years (**Figure 9B**).

## 4 Discussion

This study aimed to define a plant-architecture ideotype for soybean in the Russian Far East and to identify the genotypes closest to it. Five architectural traits and days to maturity were measured on a panel of 63 diverse accessions over three years, and multi-trait selection was applied to the resulting genotypic values. Most multi-environment studies evaluate stability in a single trait, usually yield, across a series of locations. The present study instead combined six traits into a single selection index at one site across three seasons, translating a regional breeding objective into a defined set of candidate genotypes. Architectural variation was structured along two largely independent dimensions, one of overall size and maturity and one of branching. The ideotype proved attainable only under a deliberate trait weighting, and four genotypes were selected by both indices.

### 4.1 Genetic architecture of the traits supports selection

The six traits were under moderate to strong genetic control, with broad-sense heritability ranging from 0.65 for first pod height to 0.91 for days to maturity, and the phenotypic coefficient of variation exceeded the genotypic coefficient of variation only slightly for every trait. Comparable values have been reported in soybean. Malek et al. (2014) recorded high heritability for days to maturity, plant height, branch number and pod number in a panel of soybean mutants, with narrow differences between the phenotypic and genotypic coefficients of variation, and Shilpashree et al. (2021) reported the same combination of high heritability and high genetic advance for morphological traits in vegetable soybean. The variation observed here is therefore largely heritable rather than environmentally determined, which is a prerequisite for effective selection.

Heritability alone does not indicate how much a trait can be improved, because the expected gain from selection depends on the amount of genetic variation available as well as on its heritability. It is therefore high heritability coupled with high genetic advance that identifies a trait worth selecting. On this basis the branching traits carry the highest potential for genetic improvement. The number of branches and the number of productive nodes on lateral branches combined high heritability with the largest genetic advance as a percentage of the mean, 67.3% and 78.2%, whereas days to maturity had the highest heritability of any trait but the lowest genetic advance, 15.4%. The same pattern has been reported in independent soybean panels. Malek et al. (2014) found the highest coefficients of variation and the highest genetic advance for branches per plant, 66.3% of the mean, and the lowest values for days to maturity, 11.5%, and Shilpashree et al. (2021) recorded a genetic advance of 88.8% of the mean for plant height against 20.6% for days to 50% flowering. Wang et al. (2026) likewise found that branch number in soybean is controlled by a locus with strong additive effects, which is consistent with the high genetic advance reported here. The low genetic advance for maturity, by contrast, reflects limited variation in germplasm adapted to a short growing season, where selection for earliness has already been intense. Hanson et al. (2026) report a narrow genetic base in high-latitude soybean germplasm, where ten founder cultivars account for 70.6% of the genetic contribution.

### 4.2 The independence of size and branching is genetic but not absolute

The correlation and ordination analyses showed that the six traits did not vary independently but separated into two largely independent dimensions, one of overall architectural size and one contrasting branching with the main-stem and phenological traits. The close agreement between the phenotypic and genotypic-value correlation matrices indicates that this structure is predominantly genetic in origin rather than a product of environmental covariation, and the factor analysis underlying MGIDI identified the same two-dimensional structure. A similar reduction of soybean agronomic traits to a small number of interpretable components underlies the sample-size recommendations of de Souza et al. (2023). Niu et al. (2024) reported a comparable separation in 496 accessions, in which the three branch angle traits were independent of the other architecture traits (*r* = 0.01 to 0.08), although the trait concerned there was branch angle rather than branch number.

The two dimensions have a practical consequence. The strongest single relationship, between the number of branches and the number of productive nodes on lateral branches (*r* = 0.92), identifies a branching complex that can be selected as a unit. Its near-zero correlation with the main-stem and maturity traits means branching can be improved largely independently of the size and maturity axis, so gains need not be accompanied by later maturity. This separation makes the ideotype attainable, but it is not absolute. Niu et al. (2024) identified pleiotropic loci containing *Dt1* and *Dt2* that act jointly on plant height, node number and branch number, so the phenotypic independence seen here need not hold at every locus, and correlated responses remain possible under sustained selection.

### 4.3 The central breeding tension: earliness versus architectural size

The present ideotype requires earlier maturity together with increased architectural size, and these two objectives are genetically antagonistic along the first axis of variation. In the panel, the genotypes with larger architectures were also the later-maturing ones, the same positive association between maturity and plant size documented in soybean, where flowering and maturity are commonly correlated with yield and with the vegetative growth that supports it (Copley et al., 2018). In a short-season, high-latitude environment such as the Russian Far East, varieties must reach maturity within a brief frost-free period, so early maturity is the overriding regional priority (Sinegovskaya, 2021; Zotikov and Vilyunov, 2021). Earliness, however, limits the extended vegetative development that produces a larger plant.

Adaptation to high latitudes is governed largely by allelic combinations at the maturity loci *E1–E4* (Jiang et al., 2014; Zhai et al., 2014). Jia et al. (2014) classified high-latitude varieties from northern China and the Russian Far East and found their vegetative periods to be similar whilst their reproductive periods differed, and concluded that breeding for these regions should shorten post-flowering reproductive growth whilst still ensuring sufficient vegetative accumulation. That is the balance the present ideotype seeks. A multi-trait, ideotype-based approach does not treat earliness in isolation but searches for the genotypes that reconcile it with architectural size.

The selection index made this tension quantifiable. Under equal weighting the index favoured the larger, later-maturing genotypes and moved maturity in the undesired direction, whereas prioritising earliness advanced maturity at the cost of stature. The weighting adopted represents the balance point at which earlier maturity was obtained whilst plant height and first pod height still improved. At 15% selection intensity the selected genotypes matured 3.0 days earlier than the panel mean, a selection differential of 2.3%, whilst plant height increased by 1.8 cm and first pod height by 0.2 cm, differentials of 2.9% and 2.3% (**Table 5**). The need for a deliberate weighting choice is itself a finding. It identifies the compromise achievable between earliness and size in this germplasm, and shows that the two can be combined through directed rather than unweighted selection.

### 4.4 Branching as the independent avenue of improvement

Against the constrained improvement in size and maturity, the branching traits provided a largely unconstrained source of gain. At 15% selection intensity, the selected genotypes exceeded the panel mean by 39.9% for branch number and 36.1% for productive nodes on lateral branches (**Table 5**). These values are selection differentials rather than realised responses, since no selection cycle was carried out. Neither gain was accompanied by the delay in maturity that limited the main-axis traits, because branch number and lateral-branch node number form a complex that is genetically independent of the size and maturity axis.

The breeding relevance of these gains rests on the contribution of branches and nodes to yield potential. Yield is constrained by the number of pod-bearing nodes per unit area, with pods borne on both the main stem and the branches (Kim et al., 2022). That constraint operates up to a saturation point: Egli (2013) found pod number per square metre to increase with node number per square metre only up to about 70% of the maximum node number, above which further nodes gave no additional pods. Xu et al. (2021) found pod number per plant to increase with branch number. Across their density range, however, higher branch number was associated with lower pod number per unit area and lower yield, because branching declines as density rises and their yield optimum lay at 311,000 to 350,000 plants per hectare. The branch-driven advantage appeared at the low end of their range, where branching allowed the more branched cultivar to reach its own yield maximum, whereas the less branched cultivar realised its higher potential only under dense planting. The present trials were sown at 14.3 plants per square metre, equivalent to 143,000 plants per hectare, close to the lowest density they tested. Sun et al. (2019) increased branch, node and pod number in soybean by overexpressing *GmmiR156b* and obtained a 46% to 63% increase in yield per plant; as a transgenic result this indicates the potential of the trait complex rather than a gain attainable by selection. Improving the branching complex therefore represents a gain in pod-bearing structure rather than a change in plant form alone.

### 4.5 Complementarity and convergence of the two selection indices

A genotype that approaches the ideotype on average is of limited use to a breeder if its architecture is unstable from year to year. The present design, three consecutive seasons at a single site, provided an initial assessment of this temporal dimension, and the genotype by year interaction was significant for every trait. Stability was therefore incorporated into the selection using the multi-trait stability index (MTSI) (Olivoto et al., 2019b), which is established in soybean (Abdelghany et al., 2021; Zuffo et al., 2020). MTSI does not add a stability term to the ideotype distance; it ranks genotypes on mean performance and year-to-year consistency combined, whereas MGIDI ranks them by proximity to the target architecture. Using the two together follows a strategy applied recently in soybean and other crops (Ridara et al., 2026), and distinguishes genotypes closest to the ideotype on average from those that are both close to it and consistent.

Stability-based selection improved the one trait that ideotype-distance selection did not. Under MGIDI the number of productive nodes on the main stem moved against its desired direction, decreasing by 6.1%, because the trait is genetically associated with the later maturity that selection acted against. Under MTSI the same trait increased by 1.3%. Part of the difference is procedural: MGIDI maximised proximity to the full ideotype under the size-trait weighting adopted for that index, whereas MTSI weighted all traits equally and ranked genotypes on consistent multi-trait performance. The main-stem node trait is therefore difficult to improve under strict ideotype proximity but responds favourably when selection is based on stable overall performance, which supports examining a breeding panel with more than one multi-trait index.

Because the two indices weight the traits differently, their convergence on the same four accessions indicates that these are more consistently aligned with the defined architectural ideotype than the panel as a whole (**Figure 9A**). Genotypes selected by only one index occupy intermediate positions: those chosen by MGIDI alone are close to the ideotype but less consistent across years, and those chosen by MTSI alone are consistent but architecturally further from the target. For a breeding programme with limited time and resources, this stratification provides an order of priority, advancing the dual-selected genotypes as the leading candidates whilst retaining the single-index selections as secondary material.

### 4.6 The regional ideotype: earlier, taller, and suited to mechanised harvest

The height component of the present ideotype requires justification, because plant height is a complex breeding target in soybean through its associations with maturity, lodging and yield components. Chen et al. (2024) found lodging score in a soybean recombinant inbred population to be positively correlated with flowering time, maturity time, plant height and main-stem node number, with coefficients from 0.483 to 0.783, and nine of ten major loci for those traits fell within the interval of the major lodging locus. Selecting for increased height therefore cannot be treated as independent of maturity or lodging. In the most directly comparable application of MGIDI to soybean, an early-maturing, high-yielding ideotype returned a negative selection gain for plant height of 1.91%, the only trait to move against its stated objective, height having traded off against the prioritised yield components (Maranna et al., 2021). Here the selection was instead weighted to increase plant height alongside earlier maturity.

Reduced height is favoured primarily where it confers lodging resistance, and lodging in soybean is provoked by the combination of tall stature with the dense canopies and heavy vegetative growth that accompany high-yield, high-input conditions (Hwang and Lee, 2019). Lodging pressure may be lower under the short-season, moderate-density and rainfed conditions considered here, at 14.3 plants per square metre. Increased height is also associated with more pod-bearing nodes, which links the height objective to the same productive-structure rationale that motivates the branching and node targets (Kim et al., 2022). The aim is therefore not unlimited height but an increase within the range represented in the present panel, where the tallest genotypes reached 127.6 cm. Lodging was not scored in the present trials, so where measurable lodging occurs this objective would require revision.

A second element of the ideotype, higher first pod height, addresses harvest mechanisation rather than yield directly. First pod height is a moderately heritable, architecturally determined trait that generally increases with plant height and node number (Kuzbakova et al., 2022; Jiang et al., 2018), so reduced stature lowers it. Foley et al. (1986) compared determinate and indeterminate soybean lines in the northern USA, where the determinate lines averaged 25.7 cm shorter. Their lowest pod sat 8 cm above the soil surface against 11 cm in the indeterminates, and the authors noted that with a pod length of 4 or 5 cm the lowest seed would then be only 3 to 4 cm above the ground. Selecting for shorter plants therefore risks the pod clearance on which mechanical harvest depends. The ideotype that emerges is an early-maturing plant that reaches maturity before autumn frost, carries a tall and well-branched productive structure under conditions where height is unlikely to increase lodging, and presents its lowest pods above the cutter bar.

### 4.7 Limitations

Four limitations qualify these conclusions. First, the trial was conducted at a single location over three seasons, and each year was treated as a separate environment in the MTSI analysis, so the stability assessed is year-to-year consistency under one set of site conditions rather than broad adaptation across the agro-ecological zones for which multi-location analyses such as AMMI and GGE are designed. Genotypes identified here as temporally stable would still require multi-location testing before any claim of wide adaptability. Second, three environments is a modest basis for estimating stability, and the stability parameters should be regarded as indicative rather than definitive; a larger number of seasons would estimate them more precisely. Third, the hierarchical clustering that grouped the panel into two architectural types described a moderate, largely continuous structure rather than sharply delimited groups, consistent with the quantitative nature of architectural variation, so the two groups should be interpreted as broad tendencies along the principal size axis rather than as discrete classes. Finally, the architecture traits analysed here were not accompanied by direct yield measurement, so references to productive structure throughout the Discussion denote pod-bearing capacity inferred from architecture rather than measured yield. The link between the selected architecture and realised yield, although supported by published relationships between branch and node number and yield, remains to be confirmed for these genotypes under regional conditions.

### 4.8 Breeding implications and prospects

Notwithstanding these limitations, the study provides specific guidance for soybean improvement in the region. It defines an architectural ideotype for the Russian Far East and identifies the genotypes that most nearly realise it, four accessions being close to the ideotype and temporally stable, with Gaterslebener stamm ranked first under both indices. These are usable as parents or as candidates for advancement. The branching complex is the most efficient single avenue for further architectural improvement, combining high heritability, high genetic advance and genetic independence from the earliness requirement.

More broadly, the work shows that an ideotype-based selection framework can convert a phenotypic characterisation of a germplasm panel into a defined breeding target and a ranked set of candidates, even from a single location, by treating the problem as one of identifying the best genotypes for a specified plant form rather than of mapping adaptation across space. Four steps follow. First, seed yield and its components should be measured alongside the architecture traits to test the architecture and yield link. Second, lodging should be scored, since the height component of the ideotype rests on an assumption of low lodging pressure. Third, harvest losses should be quantified under combine harvesting, distinguishing the cutter-bar losses that the first pod height objective is intended to reduce from losses due to pod shattering. Fourth, the selected genotypes should be evaluated in multi-location, multi-season trials before the four dual-selected accessions are used in crossing programmes.

## 5 Conclusion

Multi-trait selection defined a plant-architecture ideotype for soybean in the Russian Far East and identified the genotypes that most closely approach it. Five architectural traits and days to maturity were largely heritable, and the branching traits combined high heritability with the largest genetic advance, making them the most responsive component of architecture to selection. Architectural variation was structured along two largely independent dimensions, overall plant size and branching, so branching can be improved without delaying maturity. This is the finding on which the ideotype depends.

Selection towards the regional ideotype, earlier maturity combined with taller plants, a higher first pod and more branches and nodes, was attainable, but only under a deliberate trait weighting. Under equal weighting the index favoured larger, later-maturing genotypes. The antagonism between earliness and architectural size also meant that main-stem node number could not be improved alongside earliness under the ideotype-distance index, whereas the stability index improved it. Used together, MGIDI and MTSI identified four genotypes, led by Gaterslebener stamm, that combine proximity to the ideotype with year-to-year consistency.

Three conclusions follow for regional breeding. Branching is the trait complex on which to select, because it carries the most genetic variation and is not constrained by the earliness requirement. Trait weighting is not a technical detail but the step that determines whether earliness and architectural size can be combined. Gaterslebener stamm, VNIIS 1, ISZ 16 and No. 700 are the accessions to advance, as parents in crosses aimed at combining early maturity with a taller, better-branched, higher-podded plant. Because yield and lodging were not measured and all trials were at one site, these four genotypes must now be tested for seed yield, lodging and combine-harvest losses in multi-location, multi-season trials before any recommendation for release.

## Data Availability

The plot-level phenotypic data for the 63 soybean accessions and all R code used to produce the results, tables and figures presented in this study are openly available in the GitHub repository https://github.com/yawarhabib/soybean-architecture-ideotype, archived at Zenodo: https://doi.org/10.5281/zenodo.21792792.

## Supplementary material

Supplementary Figure 1 Quantile–quantile plots of the residuals from the combined analysis of variance for the six traits. Trait abbreviations are defined in **Figure 2**.

Supplementary Figure 2 Residuals against fitted values from the combined analysis of variance for the six traits. The orange line is a loess smoother. Trait abbreviations are defined in **Figure 2**.

Supplementary Figure 3 Broad-sense heritability against genetic advance as a percentage of the mean for the six traits. Trait abbreviations are defined in **Figure 2**.

Supplementary Figure 4 Cluster-validation diagnostics for the hierarchical clustering of the genotypic values: **(A)** average silhouette width and **(B)** gap statistic, for one to ten clusters. Error bars in **(B)** are standard errors from 100 bootstrap samples.
